# High Burden among Older Family Caregivers is Associated with High Prevalence of Symptoms: Data from the Swedish Study “Good Aging in Skåne (GÅS)”

**DOI:** 10.1155/2020/5272130

**Published:** 2020-07-26

**Authors:** Henrik Ekström, Nathalie Lundholm Auoja, Sölve Elmståhl, Lena Sandin Wranker

**Affiliations:** ^1^Division of Geriatric Medicine, Department of Clinical Sciences in Malmö, Lund University, Lund, Sweden; ^2^Centre for Ageing and Health, AGECAP, Department of Health and Rehabilitation, Institute of Neuroscience and Physiology, University of Gothenburg, Gothenburg, Sweden

## Abstract

**Background/Aim:**

Certain groups of informal caregivers have been shown to have worse health compared to noncaregivers. The aim of this cross-sectional study was to explore the health and gender aspects of caregiving in an older Swedish population.

**Methods:**

Our study included 5457 participants from the longitudinal, general population study “Good Aging in Skåne.” A total of 33 self-reported symptoms were obtained from questionnaires and were then divided into seven domains: depressive, musculoskeletal, gastrourinary, symptoms related to head, cardiopulmonary, symptoms related to tension, and metabolic symptoms. Multivariate logistic regression analysis was performed to assess the risk of developing symptoms in each of the seven domains, regarding caregiving burden and caregiving in relation to gender.

**Results:**

We found that caregivers, compared to noncaregivers, had a higher prevalence for depressive and tension-related symptoms. High-burden caregivers exhibited significantly more individual symptoms and a higher prevalence of symptoms in the depressive, tension, and gastrourinary domains of symptoms compared to both low-burden caregivers and noncaregivers. More than 79% of high-burden caregivers reported general fatigue, and over half of the high-burden caregivers experience depressive mood. Female caregivers showed a significantly higher risk of reporting depressive symptoms (OR = 1.54, 95% CI 1.19–1.98) and tension-related symptoms compared to male caregivers.

**Conclusion:**

Depressive and tension-related symptoms were more common in caregivers, especially in high-burden caregivers. High-burden caregivers might be at a risk of adverse mental health, and this highlights the need to offer proper support to these groups.

## 1. Introduction

With reports showing certain groups of caregivers having lower life satisfaction, higher stress levels, and impaired health compared to their noncaregiving counterparts, studying the field of elderly caregivers becomes increasingly important. Informal care is provided by nearly one-fifth of the Swedish population aged 65–80 [1], playing an important role in the Swedish health care system, as it results in reduced costs for both home help services and care facilities.

Informal caregivers are not a homogenous group, meaning not all caregiver experiences are the same. Caregiving can lead to higher life satisfaction, as some experience the responsibility of taking care of a loved one as a fulfilling task [2]. But, studies have also identified certain subgroups of informal caregivers, such as high-burden caregivers, having worse life satisfaction and reporting higher levels of stress than noncaregivers.

Female gender is a risk factor for high caregiver burden [3, 4], and female caregivers tend to report more stress and lowered life satisfaction compared to their male counterparts [5–7]. Providing extensive care, cohabiting with the care recipient, or caring for persons with dementia or cancer have also been linked with high burden [8–11].

In this paper, we will look at associations between informal caregiving and symptoms, studying participants randomly selected from a Swedish, elderly population. Self-reported life satisfaction and depressive symptoms in caregivers have been studied extensively, but there are only a few papers looking at a wider range of symptoms, both psychological and somatic, as a measure of caregiver health [8]. To our knowledge, no previous study has investigated the association between the degree of caregiver burden or burden stratified for sex and a larger number of different somatic and psychological symptoms. In our study, 23 somatic symptoms and 10 symptoms related to mental health were included.

The general aim was to investigate whether there was a difference in caregivers and noncaregivers regarding reported symptoms and if there were any differences between male and female caregivers. We theorized that the prevalence of reported symptoms would differ between caregivers and noncaregivers as well as between caregivers reporting low and high burden and between male and female caregivers.

## 2. Materials and Methods

### 2.1. Study Population

Good Aging in Skåne (GÅS) is a prospective, longitudinal, general population study, part of the Swedish National Study on Aging and Care (SNAC). Participants of GÅS are randomly selected to be tested regarding cognitive function, they are medically examined, and they have to answer a comprehensive questionnaire penetrating sociodemographic data, health and health attitudes, life circumstances, and whether they receive or offer care—formal as well as informal. Participants are then invited back for follow-up evaluations. All participants were evaluated according to the same examination protocols at both baseline assessment as well as reexamination. A more comprehensive description of the study's structure has been previously described [12, 13].

In this cross-sectional study, 8967 individuals from nine age cohorts, 60, 66, 72, 78, 81, 84, 87, 90, and >93, were invited, and 5787 (64.5%) accepted to participate. Participants were identified as caregivers based on the question: “Do you provide care to a relative or family member?” 560 participants (10.3%) were identified as currently being caregivers and providing care at least once per week, 4897 as noncaregivers, and 330 had not given an answer and were therefore excluded. The study population then consisted of 5457 participants, 2480 (45.4%) males and 2977 (54.6%) females (Table 1).

To determine caregiver burden, caregivers were asked “Do you feel strained by caregiving?” with alternative answers: “not at all,” “not particularly,” “somewhat,” “much,” and “very much.” Burden was dichotomized into high or low where high burden was defined as answering “somewhat,” “much,” and “very much” to the above question [7]. Of 560 informal caregivers, 88 reported high burden and 411 low burden, and for 61 participants, data on levels of burden were missing (Table 1).

The time spent on caregiving was assessed by the question “How often do you assist the person in need of your help?” The response options were “less than once a week,” “once a week,” “two to three times per week,” “four to six times a week,” and “every day” [7]. For 45 participants, data on time spent on caregiving were missing. Participants who provide care once a week or more often were trichotomized into “once a week,” “two to three times per week,” and “four times a week to every day” (Table 1).

Whether the care recipient received help with IADL or both IADL and PADL was assessed. IADL included movement outdoors, contact with hospitals and healthcare personnel, cooking and shopping food, transport, and managing finances. PADL included dressing and undressing, food intake, medication, wound care, and taking care of hygiene (bathing, toileting) [14]. Formal support included any assistance with IADL or PADL in accordance with the Social Services Act.

### 2.2. Assessment of Symptoms

The symptom scale used in this study was a modified version of the Gothenburg QoL instrument. The original scale was constructed during the 1970s and encompasses 30 symptoms sorted into seven categories or domains according to a previous confirmatory factor analysis. The scale has been found to have satisfactory reliability and validity with a Cronbach's alpha score ranging between 0.72 and 0.85 [15, 16]. In questionnaires, participants of the GÅS study answered if they experienced any of the 23 somatic symptoms and 10 psychological symptoms related to mental health during the past three months. The symptoms were grouped into the following seven domains: depressive symptoms, musculoskeletal symptoms, gastrointestinal- and urinary tract-related symptoms, symptoms related to head, cardiopulmonary symptoms, symptoms related to tension, and metabolic symptoms. Symptoms for the whole study population are presented under the corresponding domain in Table 2 and corresponding table stratified for sex in Table 3.

Symptoms were reported by participants by answering a four-graded scale with possible answers being “not at all,” “a little,” “somewhat,” and “a lot.” For statistical analysis, symptoms were dichotomized into “yes” if participants had experienced the symptom in question during the past 3 months and “no” if not experienced during the past 3 months. To be categorized into one or more domains of symptoms, a participant should have experienced at least one symptom of that domain during the past 3 months.

### 2.3. Assessment of Covariates

Level of education was categorized into three groups whether participants finished elementary school, high school, or university. Cohabiting status was dichotomized into cohabiting (married/cohabitant) or single (unmarried/divorced/widowed). Financial status was assessed as good or poor depending on whether the participants answered yes or no to the question “Have you had difficulties to make ends meet when it came to running expenses during the past year?” Cognitive impairment was assessed by the Mini Mental State Examination (MMSE) measuring global cognitive function. The scale range went from 0 to 30 points, and an indication of cognitive impairment was set to a score <24 points [17].

### 2.4. Statistical Analysis

Chi-square tests were used for differences of proportions between caregivers and noncaregivers and noncaregivers and low- and high-burden caregivers according to age, marital status, educational level, financial status, MMSE score, prevalence of symptoms, and domains of symptoms.

Multiple logistic regression models were constructed to assess associations between levels of caregiving and prevalence of symptoms in each of the seven domains and to assess associations between gender and prevalence of symptoms in each of the seven domains for both noncaregiver and caregiver groups (Tables 4 and 5). All regression models were adjusted for age, educational level, financial status, cohabiting status, and cognition (MMSE).

All statistical tests were two sided. A *p* value ≤0.05 was considered statistically significant. SPSS® version 24 (IBM SPSS Statistics for Windows) was used for all statistical analyses.

## 3. Ethics

The study was conducted in accordance with the Helsinki Declaration and approved by the Regional Ethics Committee at Lund University in 2002, registration no. LU 744-00. All participants provided a written consent to participate and to allow retrieval of information from the National Patient Register medical records.

## 4. Results

Looking at the characteristics of our study population, caregivers, compared to noncaregivers, were younger, more often male, more often cohabiting, had higher education, had a better financial status, and scored higher on MMSE. Among caregivers, 17.6% reported high burden and were more often females (58.0%) (Table 1). High-burden caregivers spent more time on care and a larger proportion provided both IADL and PADL compared to low-burden caregivers. In high-burden caregivers, taking care given in shared and outside own household together, 35% females and 54% males provide IADL and 65% females and 46% males provided both IADL and PADL.

Formal support was more common in high-burden caregivers (Table 1). There was no significant difference between males and females utilizing formal support, 44% in women and 41% in men.

Compared to noncaregivers, caregivers had a higher prevalence of feelings of exertion (*p* < 0.001), irritability (*p*=0.003), and being overweight (*p*=0.025). Noncaregivers, on the other hand, had a higher prevalence of difficulty walking (*p* < 0.001), appetite loss (*p*=0.024), and constipation (*p*=0.007) (Table 2).

In comparison with low-burden caregivers, high-burden caregivers had a higher prevalence for 20 of the 33 symptoms (exertion, sleeping problems, general fatigue, low spiritedness, back ache, difficulty walking, appetite loss, nausea, constipation, abdominal pain, stool and urinary incontinence, headaches, irritability, nervousness, trouble concentrating, difficulty relaxing, restlessness, weight loss, and feeling frozen) (Table 2). The number of symptoms in the high-burden group was md = 13 (*q*1 = 9.0, *q*3 = 17.2), in the low-burden group md = 8.0 (*q*1 = 4.0, *q*3 = 13.0), and in noncaregivers md = 9.0 (*q*1 = 4.0, *q*3 = 14.0) (*p* < 0.001).

Comparing male and female caregivers, females had a higher prevalence for 26 symptoms.

The number of symptoms in females was md = 10 (*q*1 = 6.0, *q*3 = 15.0) and in males md = 8.0 (*q*1 = 4.0, *q*2 = 13.0) (<0001) (Table 3).

The symptoms were grouped into seven domains, see Table 2 for summary. When analysing the prevalence of domains of symptoms in adjusted multivariate regression models, we found caregivers had significantly more depressive and tension-related symptoms than noncaregivers. Likewise, in adjusted models, we also found that depressive, gastrourinary, and tension-related symptoms were significantly more common in high-burden caregivers compared to both low-burden caregivers and noncaregivers. Additionally, high-burden caregivers exhibited more metabolic symptoms than low-burden caregivers (Table 4).

Female noncaregivers, compared to their male counterparts, reported more symptoms for all domains except for the cardiopulmonary domain. Symptoms in depressive, gastrourinary, tension related, and metabolic domains were more common in female caregivers than in male caregivers (Table 5).

## 5. Discussion

We found that caregivers more frequently than noncaregivers cohabited with someone (Table 1), and it is likely that caregivers live with the care recipient. According to the Swedish National Board of Welfare, 73% of caregivers over the age of 81 years provide care to someone in their own household [1], and our results are in line with those numbers. High-burden caregivers also cohabited more than low-burden caregivers. This was not statistically significant but can possibly be attributed to a power problem as it has previously been shown that those living with the individual receiving care report higher burden and stress [3, 18].

It may seem surprising that a slightly larger proportion of caregivers were men, but family elderly care is less gendered in Sweden. Among caregivers 75 years and above, there is no major difference between women's and men's caregiving in terms of time or frequency. However, there is a difference in the fact that men provide less burdensome care, practical help, and financial support (IADL), while women take a greater part in more demanding tasks such as supervision and personal care (PADL). This is in line with the findings in this study where a larger proportion of females, compared to males, provided both IADL and PADL and experience caregiving as burdensome [1, 19].

Older individuals show a decline in health and function and are more prone to exhibiting symptoms like joint pains, sleeping problems, headaches, and poor appetite [20]. In our material, caregivers were slightly younger than noncaregivers, while high-burden caregivers were slightly older than low-burden caregivers (Table 1). We suspected that the slight differences in age would in part act as an explanation to the higher prevalence of symptoms in high-burden caregivers (Table 2). However, when adjusted for age in multivariate regression models, we still saw a higher prevalence of symptoms in both caregivers compared to noncaregivers, as well as in high-burden caregivers compared to low-burden caregivers (Table 4).

It is mandatory for Swedish municipalities to offer formal care to sick individuals, while the same does not apply for many other countries. Comparing 19 European countries, Sweden had the highest degree of availability of formal care [21]. Outside of Europe, formal support is even more scarce [22]. Availability of formal care has been shown to mitigate effects of stressors and improve wellbeing in caregivers [23, 24]. Therefore, it was surprising to find that the prevalence of depressive and tension-related symptoms was so high in our Swedish caregiver population. Depressive mood was reported by 27.6% of the male caregivers and 39.2% of the females (data not shown), which should be compared to a Belgian study looking at caregivers to frail, elderly people where 11–22% of caregivers reported feelings of depression and anger [25], or a Norwegian study where 18% male and 30% female caregivers to cancer patients reported depressive symptoms [26].

A study on caregivers from North Carolina, US, showed those giving care to individuals with chronic illness reported high levels of fatigue concepts of burnout (59%) [27]. In our study population, a staggering 66.8% of all caregivers and 79.1% of high-burden caregivers reported experiencing general fatigue during the past three months (Table 2). It could be speculated that a wider definition of high-burden caregivers might have been used in the North Carolina study, as 71% of their study population reported caregiver burden, while a comparatively low proportion (16%) of our study population reported high burden. It is possible that factors such as social support and coping mechanisms could explain the differences between these studies, as they are protective factors against high caregiver burden [3].

In comparison with low-burden caregivers, not only did we find that high-burden caregivers reported a higher prevalence for 20 of the 33 listed symptoms, we also found that there was a substantially higher proportion of high-burden caregivers experiencing individual symptoms as well as domains of symptoms (Tables 2 and 4). High caregiver burden has in earlier studies been shown to be correlated with lower life satisfaction, stress, and depressive symptoms [7, 28], and our results are supported by these findings. High-burden caregivers also tend to have worse self-reported somatic health [29, 30]. An interesting finding though was that there were no significant differences regarding the somatic domains of symptoms between noncaregivers and caregivers and that proportions with symptoms in low-burden caregivers were lower in 6 out of 7 domains compared to noncaregivers (Table 4). This could be explained by the fact that those with a low burden were already in better health from the beginning or that caregiving that is less stressful can create a sense of meaningfulness which in turn can be conducive to health [2].

High-burden caregivers had a higher prevalence of gastrointestinal and urinary tract symptoms than noncaregivers and more so than low-burden caregivers. Looking at individual symptoms, high-burden caregivers had a higher prevalence of appetite loss and weight loss (Table 2). Poor nutritional status in the elderly has been linked to lower life satisfaction and depression, and depression could in turn lead to poor nutritional status due to anhedonia and appetite loss [31, 32]. It has been shown that gastrointestinal symptoms are associated with stress and depression [33], and it is likely in our study population to be an expression of high stress levels.

Female sex is a risk factor not only for becoming a caregiver but also for experiencing caregiving as a burden [3, 22]. High caregiver burden is in turn linked with poor caregiver health. In regression models, we found that both female noncaregivers and female caregivers reported more symptoms than their male counterparts. Female noncaregivers had a higher prevalence than male noncaregivers in six of the seven domains of symptoms, and female caregivers had a higher prevalence in four of the seven domains compared to male caregivers. Between female and male caregivers, the differences in proportions in the domains of symptoms are ranging from 2.3% to 13.6% (Table 5). Again, we saw that not only was there a difference between the groups, but also that the difference in proportions was substantial.

The gender differences found in our study population could be explained by physiological and social aspects. In a caregiving context, coping mechanisms, social support, and socioeconomic factors have all been shown to affect perceived burden [22, 25]. Gender and socioeconomic inequalities were reported from a Spanish nationwide survey [21] where women had less access to formal support. In our study, there was no significant difference between male and female caregivers when it came to formal support with IADL and PADL, but it should be noted that only about half of both female and male caregivers received any formal support.

We have previously reported from the Good Aging in Skåne project that caregiver burden differs depending on the main illness of the care recipient, with higher burden related to individuals with depression and dementia. Formal support was, however, offered only to 23% of caregivers to those with dementia, but to 77% of caregivers to fractured individuals [8]. Diagnosis of the care recipient and their possible effects on reported symptoms in caregivers, as well as the relationship between the caregiver and the care recipient, other than that the care recipient is a relative or family member, are however aspects that we have not explored in this paper and should be theories of future studies.

Men have in multiple studies been found to be downplaying pain or symptoms as well as exhibiting reluctance in seeking health care when needed [34, 35]. One theory explaining this behaviour is a willingness—conscious or not—to adhere to an image of masculinity where being sick or in pain is seen as weakness. This theory could especially be held true for this study population as the participants are from older generations where expectations on gender roles were more stringent than they are today and could be a possible explanation to the differences we found between males and females in our study population.

A strength of our study is our large study population, randomly selected from the Swedish National Population Registry. We only included caregivers currently giving care and providing care at least once a week, as there is emerging evidence that the frequency of caregiving is related to caregiver health and caregiver burden [36].

A possible limitation is the low proportion of caregivers. Compared to numbers from the Swedish Board of Health and Welfare, where 20% of the general population reported being a caregiver and 15% reported that they provided care at least once a week, only 10.3% were caregivers in our study population [1]. At baseline assessment, the participation rate was around 60%. In-home assessments were offered to include participants that might be too frail, or otherwise unable to leave home. However, it is possible that those excluded are caregivers giving extensive care, or with relatives too sick and frail to be left alone for longer periods of time. In addition, more than one-fifth of caregivers were over 80 years old, and it cannot be ruled out that for health reasons they were unable to attend. This could mean our results are an underestimation of the number of caregivers and true symptom burden.

It could be seen as a limitation that we in this study identified caregivers and their respective levels of caregiving burden using a single-item question. On the contrary, it can be advantageous to use a single question as it is easily accepted by the participants and that the meaning of the question is presented directly. The questions we used to identify caregivers and levels of burden have shown validity in a previous study by us where caregiving itself and levels of burden were associated with health-related quality of life [7].

## 6. Conclusion

This study shows that there are differences in symptoms reported by caregivers and noncaregivers, as well as between male and female caregivers. Depressive, tension related, gastrourinary, and metabolic symptoms were more prevalent in high burden and female caregivers. More demanding care including assistance with PADLs was more common in high burden and female caregivers, while only half of the participants in both these groups utilized the opportunities for formal municipal support. These results highlight the importance of formal support and that formal support should be offered to all informal caregivers in need of assistance, especially to high burden and female caregivers, as these groups might be at risk for a poor mental and somatic health.

## Figures and Tables

**Table 1:** Characterization of the study population, comparing caregivers and noncaregivers, as well as comparing caregivers reporting high burden and low burden.

Variables *n* (%)	Caregivers 560 (10.3)	Noncaregivers 4897 (89.7)	*p*	High burden 88 (17.6)	Low burden 411 (82.4)	*p*
Sex						
Male	285 (50.9)	2195 (44.8)	0.006	37 (42.0)	222 (54.0)	0.041
Female	275 (49.1)	2702 (55.2)		51 (58.0)	189 (46.0)	
Age, decades						
60–69	390 (69.6)	2993 (69.6)	<0.001	53 (60.2)	287 (69.8)	0.201
70–79	44 (7.9)	484 (9.9)		10 (11.4)	32 (7.8)	
80+	126 (22.5)	1420 (29.0)		25 (28.4)	92 (22.4)	
Age, years (mn) (sd)	67.5 (9.4)	69.4 (10.3)	<0.001	69.1 (9.9)	67.5 (9.4)	0.159
Education						
Elementary	174 (34.1)	2136 (45.4)	<0.001	32 (36.8)	137 (33.8)	0.605
Secondary	170 (33.3)	1425 (30.3)		25 (28.7)	139 (34.3)	
University	167 (32.7)	1145 (24.3)		30 (34.5)	129 (31.8)	
Cohabiting status						
Cohabiting	421 (75.2)	2808 (57.4)	<0.001	71 (80.7)	302 (73.5)	0.362
Single	139 (24.8)	2085 (42.6)		17 (19.3)	109 (26.5)	
Financial status						
Good	482 (94.1)	4443 (94.5)	0.712	81 (92.0)	383 (94.6)	0.597
Poor	30 (5.9)	257 (5.5)		7 (8.0)	22 (5.4)	
MMSE						
≥24	447 (93.3)	4053 (90.0)	0.024	74 (88.1)	373 (94.4)	0.035
<24	32 (6.7)	447 (10.0)		10 (11.9)	22 (5.6)	
Time spent on caregiving						
Once a week	197 (39.5)	—		16 (18.2)	183 (44.5)	<0.001
2-3 times a week	96 (19.2)	—		16 (18.2)	76 (18.5)	
4–7 times a week	206 (41.3)	—		56 (63.6)	152 (37.0)	
IADL shared household	119 (23.3)	—		12 (13.6)	107 (26.0)	<0.001
IADL outside household	228 (44.6)	—		26 (29.5)	193 (47.0)	0.001
IADL/PADL shared household	105 (20.5)	—		35 (39.8)	70 (17.0)	<0.001
IADL/PADL outside household	59 (11.5)	—		15 (17.0)	41 (10.0)	0.001
Formal support	73 (13.0)	—		46 (52.3)	26 (6.3)	<0.001

*Note*. Missing data: high or low burden *n* = 61, time spent on caregiving *n* = 61, and ADLs *n* = 49.

**Table 2:** Prevalence of symptoms *n* (%) comparing noncaregivers and caregivers, as well as stratified for high-burden caregivers and caregivers reporting low burden.

Symptoms, *n* (%)	Caregivers 560 (10.3%)	Noncaregivers 4897 (89.7%)	*p*	High-burden caregivers 88 (17.6%)	Low-burden caregivers 411 (82.4%)	*p*
Depressive symptoms						
Feeling exerted	172 (31.3)	912 (19.2)	<0.001	51 (59.3)	106 (26.3)	<0.001
Sleeping problems	228 (41.6)	2060 (43.4)	0.420	52 (59.3)	154 (38.3)	<0.001
General fatigue	366 (66.8)	3142 (66.1)	0.764	68 (79.1)	268 (66.7)	0.024
Depressive mood	200 (36.4)	1615 (34.0)	0.253	49 (57.0)	138 (34.2)	<0.001
Tearfulness	179 (32.6)	1558 (32.8)	0.945	36 (41.9)	127 (31.5)	0.065

Musculoskeletal symptoms						
Joint pains	262 (47.7)	2389 (50.3)	0.262	42 (48.8)	193 (47.9)	0.873
Back ache	284 (51.7)	2486 (52.3)	0.799	53 (61.6)	201 (49.9)	0.048
Leg pains	239 (46.1)	2324 (50.2)	0.098	47 (54.7)	180 (45.3)	0.117
Difficulty walking	125 (24.1)	1500 (32.3)	<0.001	34 (39.5)	85 (21.4)	<0.001

Gastrourinary symptoms						
Appetite loss	42 (7.7)	512 (10.8)	0.024	12 (14.0)	23 (5.7)	0.007
Nausea	53 (9.7)	479 (10.1)	0.753	13 (15.1)	32 (7.9)	0.037
Diarrhoea	65 (11.8)	550 (11.6)	0.850	13 (15.1)	44 (10.9)	0.271
Constipation	63 (11.5)	756 (15.9)	0.007	19 (22.4)	41 (10.2)	0.002
Abdominal pain	111 (20.3)	994 (20.9)	0.718	24 (27.9)	74 (18.4)	0.046
Difficulty urinate	56 (10.2)	471 (9.9)	0.819	13 (15.1)	40 (10.0)	0.162
Incontinence-urine	97 (18.7)	983 (21.1)	0.202	25 (29.1)	66 (16.6)	0.007
Incontinence-stool	32 (6.2)	267 (5.7)	0.682	13 (15.1)	16 (4.0)	<0.001

Symptoms related to head						
Headaches	153 (29.6)	1232 (25.9)	0.132	34 (39.5)	106 (26.8)	0.018
Dizziness	134 (25.9)	1267 (27.3)	0.498	26 (30.2)	97 (24.4)	0.263
Auditory problems	172 (33.2)	1614 (35.1)	0.488	32 (37.2)	128 (32.2)	0.375
Eye problems	155 (30.0)	1560 (33.6)	0.101	32 (37.6)	117 (30.1)	0.139

Cardiopulmonary symptoms						
Chest pains	91 (16.6)	742 (15.6)	0.514	20 (23.3)	63 (16.3)	0.089
Breathlessness	157 (30.3)	1528 (32.9)	0.239	32 (37.2)	117 (29.7)	0.159
Cough	153 (27.9)	1330 (28.0)	0.960	30 (34.9)	107 (26.6)	0.118

Symptoms related to tension						
Irritability	295 (53.7)	2240 (47.1)	0.003	61 (70.9)	207 (51.4)	0.001
Nervousness	182 (33.2)	1508 (31.7)	0.502	43 (50.0)	124 (30.8)	0.001
Trouble concentrating	205 (37.3)	1775 (37.3)	0.998	50 (58.1)	146 (36.2)	<0.001
Difficulty relaxing	236 (43.0)	1904 (40.1)	0.190	60 (69.8)	155 (38.5)	<0.001
Restlessness	180 (32.8)	1502 (31.6)	0.578	44 (51.2)	115 (28.5)	<0.001

Metabolic symptoms						
Weight loss	30 (5.5)	339 (7.1)	0.145	9 (10.5)	17 (4.2)	0.019
Overweight	247 (45.0)	1904 (40.0)	0.025	45 (52.3)	182 (45.2)	0.227
Feeling frozen	117 (22.6)	1234 (26.5)	0.054	30 (34.9)	80 (20.2)	0.003
Sweats	148 (28.6)	1249 (26.2)	0.409	26 (30.2)	110 (27.7)	0.637

No. of symptoms md (*q*1, *q*3)	9.0 (4.0–14.0)	9.0 (5.0–14.0)	0.806	13.0 (9.0–17.2)	8.0 (4.0–13.0)	<0.001

**Table 3:** Prevalence of symptoms *n* (%) comparing male and female caregivers.

Symptoms, *n* (%)	Male caregivers 285 (50.9%)	Female caregivers 275 (49.1%)	*p*
Depressive symptoms			
Feeling exerted	74 (26.6)	98 (36.2)	0.016
Sleeping problems	88 (31.8)	140 (51.7)	<0.001
General fatigue	180 (64.7)	186 (68.9)	0.304
Depressive mood	87 (31.3)	113 (41.7)	0.011
Tearfulness	57 (20.5)	122 (45.0)	<0.001

Musculoskeletal symptoms			
Joint pains	129 (46.4)	133 (49.1)	0.530
Back ache	138 (49.6)	146 (53.9)	0.321
Leg pains	115 (43.2)	124 (49.2)	0.173
Difficulty walking	66 (24.8)	59 (23.4)	0.710

Gastrourinary symptoms			
Appetite loss	13 (4.7)	29 (10.7)	0.008
Nausea	22 (7.9)	31 (11.4)	0.162
Diarrhoea	36 (12.9)	29 (10.7)	0.415
Constipation	25 (9.0)	38 (14.1)	0.062
Abdominal pain	51 (18.3)	60 (22.2)	0.259
Difficulty urinate	48 (17.3)	8 (3.0)	<0.001
Incontinence-urine	40 (15.0)	57 (22.6)	0.027
Incontinence-stool	16 (6.0)	16 (6.4)	0.865

Symptoms related to head			
Headaches	55 (20.8)	98 (38.9)	<0.001
Dizziness	59 (22.2)	75 (29.8)	0.049
Auditory problems	97 (36.5)	75 (29.8)	0.105
Eye problems	79 (29.8)	76 (30.2)	0.931

Cardiopulmonary symptoms			
Chest pains	50 (18.1)	41 (15.1)	0.358
Breathlessness	82 (30.8)	75 (29.8)	0.792
Cough	70 (25.2)	83 (30.6)	0.155

Symptoms related to tension			
Irritability	143 (51.4)	152 (56.1)	0.275
Nervousness	76 (27.3)	106 (39.1)	0.003
Trouble concentrating	96 (34.5)	109 (40.2)	0.168
Difficulty relaxing	99 (35.6)	137 (50.6)	<0.001
Restlessness	90 (34.2)	90 (33.2)	0.835

Metabolic symptoms			
Weight loss	15 (5.4)	15 (5.5)	0.943
Overweight	54 (20.4)	63 (25.0)	0.016
Feeling frozen	117 (22.6)	1234 (26.5)	0.209
Sweats	54 (20.3)	94 (37.3)	<0.001

No. of symptoms md (*q*1, *q*3)	8.0 (4.0–13.0)	10.0 (6.0–15.0)	<0001

**Table 4:** Multiple logistic regression models for domains of symptoms, comparing caregivers with noncaregivers (reference), high-burden caregivers with low-burden caregivers (reference), and high-burden caregivers with noncaregivers (reference).

	Caregivers vs. noncaregivers (ref.)				Caregivers, high burden vs. low burden (ref.)				Caregivers, high burden vs. noncaregivers (ref.)			
Domains of symptoms, *n* (%)	CG *n* = 560	NCG *n* = 4897	OR	CI 95%	CHB *n* = 88	CLB *n* = 411	OR	CI 95%	CHB *n* = 88	NCG *n* = 4897	OR	CI 95%
Depressive	440 (80.4)	3781 (79.5)	**1.35**	1.05–1.73	82 (95.3)	318 (79.3)	**5.02**	1.76–14.37	82 (95.3)	440 (80.4)	**5.26**	1.91–14.50
Musculoskeletal	395 (73.4)	3565 (75.5)	0.96	0.77–1.19	69 (80.2)	285 (71.3)	1.42	0.79–2.56	69 (80.2)	395 (73.4)	1.30	0.75–2.26
Gastrourinary	272 (51.6)	2471 (51.5)	1.13	0.93–1.37	61 (70.9)	185 (46.5)	**2.41**	1.42–4.08	61 (70.9)	272 (51.6)	**2.36**	1.44–3.84
Head related	326 (63.1)	3023 (65.1)	1.06	0.86–1.30	64 (74.4)	237 (59.8)	**1.76**	1.01–3.09	64 (74.4)	326 (63.1)	1.61	0.96–2.71
Cardiopulmonary	260 (49.6)	2333 (49.9)	1.12	0.93–1.36	45 (52.3)	195 (49.0)	1.10	0.67–1.80	45 (52.3)	260 (49.6)	1.14	0.73–1.78
Tension related	403 (73.4)	3290 (69.2)	**1.35**	1.08–1.67	78 (90.7)	283 (70.2)	**3.76**	1.74–8.13	78 (90.7)	403 (73.4)	**4.12**	2.00–8.60
Metabolic	351 (66.7)	3044 (65.1)	1.20	0.98–1.47	66 (76.7)	255 (64.1)	1.64	0.93–2.88	66 (76.7)	351 (66.7)	**1.78**	1.06–2.98

*Note*. Significant ORs are in bold, *p* < 0.05. All regression models are adjusted for sex, age, education level, cohabiting status, MMSE, and financial status.

**Table 5:** Multiple logistic regression models for domain of symptoms, comparing male noncaregivers (reference) with female noncaregivers and male caregivers (reference) with female caregivers.

	Male noncaregivers (ref.) vs female noncaregivers				Male caregivers (ref.) vs female caregivers			
Domain of symptoms, *n* (%)	Male NCG *n* = 2195	Female NCG *n* = 2702	OR	CI 95%	Male CG *n* = 285	Female CG *n* = 277	OR	CI 95%
Depressive	1557 (73.2)	2224 (84.7)	**1.36**	1.26–1.47	210 (75.8)	230 (85.2)	**1.54**	1.19–1.98
Musculoskeletal	1509 (71.6)	2056 (78.6)	**1.18**	1.10–1.26	195 (71.4)	200 (75.5)	1.14	0.93–1.40
Gastrourinary	953 (45.4)	1464 (56.5)	**1.19**	1.12–1.27	125 (46.5)	147 (57.0)	**1.24**	1.03–1.50
Head related	1248 (59.9)	1775 (69.2)	**1.18**	1.10–1.25	160 (60.4)	166 (65.9)	1.14	0.94–1.39
Cardiopulmonary	1012 (48.3)	1321 (51.1)	1.02	0.96–1.09	130 (48.5)	130 (50.8)	1.02	0.85–1.23
Tension related	1379 (64.7)	1911 (69.2)	**1.18**	1.10–1.26	189 (68.0)	214 (79.0)	**1.26**	1.02–1.56
Metabolic	1189 (56.7)	1855 (71.8)	**1.37**	1.28–1.46	163 (60.1)	188 (73.7)	**1.33**	1.09–1.62

*Note*. Significant ORs are in bold, *p* < 0.05. All regression models are adjusted for age, education level, cohabiting status, MMSE, and financial status.

## Data Availability

The authors confirm that the data supporting the findings of this study are available within the article.

## References

[1] The National Board of Health and Welfare, Sweden, 2012, https://www.socialstyrelsen.se/publikationer2012/2012-8-15

[2] Weisser F. B., Bristowe K., Jackson D. (2015). Experiences of burden, needs, rewards and resilience in family caregivers of people living with Motor Neurone disease/Amyotrophic Lateral Sclerosis: a secondary thematic analysis of qualitative interviews. *Palliative Medicine*.

[3] Adelman R. D., Tamanova L. L., Delgado D., Dion S., Lachs M. S. (2014). Caregiver burden: a clinical review. *JAMA*.

[4] Gresswell I., Lally L., Adamis D., McCarthy G. M. (2018). Widening the net: exploring social determinants of burden of informal carers. *Irish Journal of Psychological Medicine*.

[5] Pinquart M., Sorensen S. (2006). Gender differences in caregiver stressors, social resources, and health: an updated meta-analysis. *The Journals of Gerontology Series B: Psychological Sciences and Social Sciences*.

[6] Yee J. L., Schulz R. (2000). Gender differences in psychiatric morbidity among family caregivers: a review and analysis. *The Gerontologist*.

[7] Dahlrup B., Ekström H., Nordell E., Elmståhl S. (2015). Coping as a caregiver: a question on life satisfaction and health-related quality of life. *Archives of Gerontology and Geriatrics*.

[8] Elmståhl S., Dahlrup B., Ekström H., Nordell E. (2018). The association between medical diagnosis and caregiver burden: a cross-sectional study of recipients of informal support and caregivers from the general population study “good aging in Skåne,” Sweden. *Aging Clinical Experimental Research*.

[9] Danacı E., Koç Z. (2018). Caregiving burden and life satisfaction among caregivers of cancer patients admitted to the emergency department. *Clinical Nursing Research*.

[10] Li Q. P., Mak Y. W., Loke A. Y. (2013). Spouses’ experience of caregiving for cancer patients: a literature review. *International Nursing Review*.

[11] Chiao C.-Y., Wu H.-S., Hsiao C.-Y. (2015). Caregiver burden for informal caregivers. *International Nursing Review*.

[12] Lagergren M., Fratiglioni L., Hallberg I. R. (2004). A longitudinal study integrating population, care and social services data. The Swedish National study on Aging and Care (SNAC). *Aging Clinical and Experimental Research*.

[13] Ekström H., Elmståhl S. (2006). Pain and fractures are independently related to lower walking speed and grip strength: results from the population study “good ageing in Skåne”. *Acta Orthopaedica*.

[14] Sonn U., Asberg K. H. (1991). Assessment of activities of daily living in the elderly. A study of a population of 76-year-olds in Gothenburg, Sweden. *Scandinavian Journal of Rehabilitation Medicine*.

[15] Sullivan M., Karlsson J., Bengtsson C., Furunes B., Lapidus L., Lissner L. (1993). «The göteborg quality of life instrument» -a psychometric evaluation of assessments of symptoms and well-being among women in a general population. *Scandinavian Journal of Primary Health Care*.

[16] Tibblin G., Bengtsson C., Furunes B., Lapidus L. (1990). Symptoms by age and sex. *Scandinavian Journal of Primary Health Care*.

[17] Folstein M. F., Folstein S. E., McHugh P. R. (1975). Mini-mental state. A practical method for grading the cognitive state of patients for the clinician. *Journal of Psychiatric Research*.

[18] Kim H., Chang M., Rose K., Kim S. (2012). Predictors of caregiver burden in caregivers of individuals with dementia. *Journal of Advanced Nursing*.

[19] Colombo F., Llena-Nozal A., Mercier J., Tjadens F. (2011). *Help Wanted? Providing and Paying for Long-Term Care*.

[20] Enkvist Å., Ekström H., Elmståhl S. (2012). Life satisfaction and symptoms among the oldest-old: results from the population study called good ageing in Skåne. *Archives of Gerontology and Geriatrics*.

[21] Verbakel E. (2018). How to understand informal caregiving patterns in Europe? The role of formal long-term care provisions and family care norms. *Scandinavian Journal of Public Health*.

[22] Abajo M., Rodríguez-Sanz M., Malmusi D., Salvador M., Borrell C. (2017). Gender and socio-economic inequalities in health and living conditions among co-resident informal caregivers: a nationwide survey in Spain. *Journal of Advanced Nursing*.

[23] Verbakel E., Metzelthin S. F., Kempen G. I. J. M. (2018). Caregiving to older adults: determinants of informal caregivers’ subjective well-being and formal and informal support as alleviating conditions. *The Journals of Gerontology Series B: Psychological Sciences and Social Sciences*.

[24] Hartmann M. L., Wens J., Verhoeven V., Remmen R. (2012). The effect of caregiver support interventions for informal caregivers of community-dwelling frail elderly: a systematic review. *International Journal of Integrated Care*.

[25] Hartmann M. L., Mello J. D. A., Anthierens J. (2019). Caring for a frail older person: the association between informal caregiver burden and being unsatisfied with support from family and friends. *Age and Ageing*.

[26] Grov E. K., Dahl A. A., Moum T., Fosså S. D. (2005). Anxiety, depression, and quality of life in caregivers of patients with cancer in late palliative phase. *Annals of Oncology*.

[27] Lynch S. H., Shuster G., Lobo M. L. (2018). The family caregiver experience–examining the positive and negative aspects of compassion satisfaction and compassion fatigue as caregiving outcomes. *Aging & Mental Health*.

[28] Kim D. (2017). Relationships between caregiving stress, depression, and self-esteem in family caregivers of adults with a disability. *Occupational Therapy International*.

[29] Berglund E., Lytsy P., Westerling R. (2015). Health and wellbeing in informal caregivers and non-caregivers: a comparative cross-sectional study of the Swedish general population. *Health and Quality of Life Outcomes*.

[30] Musich S., Wang S. S., Kraemer S., Hawkins K., Wicker E. (2017). Caregivers for older adults: prevalence, characteristics, and health care utilization and expenditures. *Geriatric Nursing*.

[31] Ghimire S., Baral B. K., Karmacharya I., Callahan K., Mishra S. R. (2018). Life satisfaction among elderly patients in Nepal: associations with nutritional and mental well-being. *Health and Quality of Life Outcomes*.

[32] Vafaei Z., Mokhtari H., Sadooghi Z., Meamar R., Chitsaz A., Moeini M. (2013). Malnutrition is associated with depression in rural elderly population. *Journal of Research in Medical Sciences: The Official Journal of Isfahan University of Medical Sciences*.

[33] Locke G. R., Weaver A. L., Melton L. J., Talley N. J. (2004). Psychosocial factors are linked to functional gastrointestinal disorders: a population based nested case-control study. *American Journal of Gastroenterology*.

[34] Galdas P. M., Cheater F., Marshall P. (2005). Men and health help-seeking behaviour: literature review. *Journal of Advanced Nursing*.

[35] Courtenay W. H. (2000). Constructions of masculinity and their influence on men’s well-being: a theory of gender and health. *Social Science & Medicine*.

[36] Andren S., Elmståhl S. (2008). The relationship between caregiver burden, caregivers´ perceived health, and their sense of coherence in caring for elders with dementia. *Journal of Clinical Nursing*.

